# Human eyelid behavior is driven by segmental neural control of the orbicularis oculi

**DOI:** 10.1073/pnas.2508058122

**Published:** 2025-08-07

**Authors:** Jinyoung Kim, Ashley Shirriff, Jordan N. Cornwell, Maria Paula Quintero Mutis, Ereni Delis, Sophia Wang, Daniel B. Rootman, Tyler R. Clites

**Affiliations:** ^a^Department of Mechanical and Aerospace Engineering, University of California Los Angeles, Los Angeles, CA 90095; ^b^Department of Ophthalmic Plastic and Reconstructive Surgery, University of California Los Angeles, Los Angeles, CA 90095

**Keywords:** eyelid, neural control of movement, biomechanics, orbicularis oculi, electromyography

## Abstract

The eyelid performs critical functions to protect the eye and preserve functional vision. These functions are driven by contraction of the orbicularis oculi (OO), which is a unique skeletal muscle with a circular geometry and diffuse innervation. It is thought that this distributed innervation may allow for differential segmental activation and contraction, but it is not currently understood how sequenced activation patterns relate to differential muscle contraction, nor how segmental contraction creates the kinematics that drive the eyelid’s critical functions. In fact, motion of the eyelid has predominantly been modeled in only a single dimension (open–close). Here, we show that eyelid motion has important two-dimensional features that vary between eyelid behaviors. Using distributed intramuscular electromyography, we further show that activation differs segmentally across the OO, and that patterns of activation change to produce different behavior-specific eyelid kinematics. Our results demonstrate the role of segmental activation in eyelid motion, highlighting the importance of precise neural control in producing natural eyelid behavior. We anticipate that this research is a starting point for robust mechanistic models of eyelid function. This knowledge has critical implications for diagnosis and treatment of eyelid paralysis.

## Significance

The human eyelid is an intricate structure that plays a critical role in maintaining vision, but its neuromuscular control remains poorly understood. Here, we present high-resolution intramuscular recordings of muscle activation from the orbicularis oculi, coupled with precise measurements of eyelid movement, during different behaviors. Our results show that eyelid motion is complex, behavior-specific, and driven by precise sequences of muscle activation. These findings challenge simplistic models of eyelid function and highlight the importance of fine neural control in generating natural eyelid motion. This work lays the foundation for improved diagnosis of eyelid pathology, as well as future development of neuroprosthetic devices to restore eyelid function.

The human eyelid protects the eye and maintains functional vision (1). Eyelid movement is driven by the orbicularis oculi (OO) muscle, which has a unique circular structure (2, 3) and a remarkably diffuse pattern of innervation (4–6). This diffuse innervation is thought to allow for spatiotemporal differentiation in activation of different parts of the OO (3, 7), which in turn enables nuanced differential motion of the gross eyelid structure. It is not currently understood how sequenced segmental activation and muscle excursion of the OO produces functionally distinct eyelid kinematics during different eyelid behaviors.

Humans have evolved to rely on a diverse set of eyelid behaviors to protect the eye, preserve vision, and communicate emotion. Spontaneous and voluntary blink, which are the most frequent eyelid behaviors, are responsible for moisturizing and lubricating the cornea by spreading three distinctive layers of mucous, aqueous, and oily layers of tear film across the ocular surface (2, 8, 9). This function is crucial to preventing corneal abrasion, infection, and dryness, all of which are incompatible with functional vision. Spontaneous and voluntary blink also facilitate tear pumping, which serves to clear liquid and debris from the ocular surface by pushing tears medially across the eye and into the lacrimal sac (10–12). Reflexive blink (rapid and transient) and forced closure (forceful and sustained) both play a critical role in protecting the cornea from physical threat (2). Soft closure blocks unwanted light, helps to relax the eye, and can restore moisture to a dry eye (e.g., after prolonged screen usage) (13). Eyelids also serve an important social-cultural purpose, enabling complex emotional expression (e.g. squinting, smiling, and winking) (8).

The eyelid moves differently to serve the specific functions associated with each behavior (14). Because motion of the eyelid is governed predominantly by contraction of the OO and the levator palpebrae superioris (LPS), these differential eyelid *kinematics* must be driven by changes in muscle *activations*. For instance, it has been shown that eyelid closing velocities differ between spontaneous, voluntary, and reflexive blinks (15–17). As evidence of what may drive these differences, correlations have been found between OO activation and maximum closing velocity (15), and between OO EMG duration and closing-phase blink duration during spontaneous and voluntary blinks (16). Recent studies have also suggested that differential eyelid behaviors may be driven by segmental, localized activation of the OO (7): The “intercanthal” segment is thought to serve blink, wetting, and tear pump, whereas the “extracanthal” segment is responsible for squeezing and rapid closure. However, the temporospatial resolution of these studies has been insufficient to capture segmental activation patterns, and it has not been possible to link segmental activation to either segmental excursion of the OO muscle or the resultant kinematics of the full eyelid.

Thus far, eyelid motion studies have focused predominantly on a single dimension (closure and its time derivatives), providing a limited picture of each behavior. Several different technologies have been used to track the eyelid, including magnetic search coils (14–16, 18–20), gyroscopes (21), high-speed video (22–26), and infrared marker tracking (23, 27); in all of these cases, tracking has been limited in resolution to only one or two points on the eyelid, typically in the center of the upper eyelid margin and often only in vertical displacement In two notable exceptions, it has been demonstrated that blinking and gaze shift include both vertical and horizontal movements of the upper and lower eyelids (28, 29). With this limited kinematic information, it has not been possible to identify how the eyelid moves differently to satisfy the different functional goals of each behavior. Low temporospatial resolution of EMG recording has also limited our ability to capture differences in segmental OO activation patterns between behaviors. Previous efforts to study eyelid activation have used a relatively small number of surface EMG electrodes across the surface of the eyelid (14, 30–32) and have lacked the resolution and density to capture segmental EMG from the pretarsal portion of the OO, which is where the most nuanced and critical activation patterns are thought to occur (7, 33). Concentric needle EMG electrodes (33, 34) have been used to provide more precise EMG measurements than is possible with surface EMG; however, these electrodes were limited in density and location due to safety concerns around blinking with needles in the eyelid.

In this study, we sought to address these limitations by studying eyelid neuromechanics with high spatial and temporal resolution, via segmental intramuscular EMG and three-dimensional motion capture from markers along the eyelid margin. Our primary objective was to elucidate the differential activation patterns and resultant kinematics associated with each eyelid behavior, as a critical step toward mechanistically linking those neuromechanics to eyelid function. We also explored muscle *excursion* in the pretarsal OO, by tracking motion of the eyelid skin. These investigational tools have led us to findings about kinematic characteristics and temporospatial EMG activation patterns across different eyelid behaviors.

## Results

### Eyelid Motion Differs in At Least Two Dimensions between Behaviors.

**Fig. 1.**
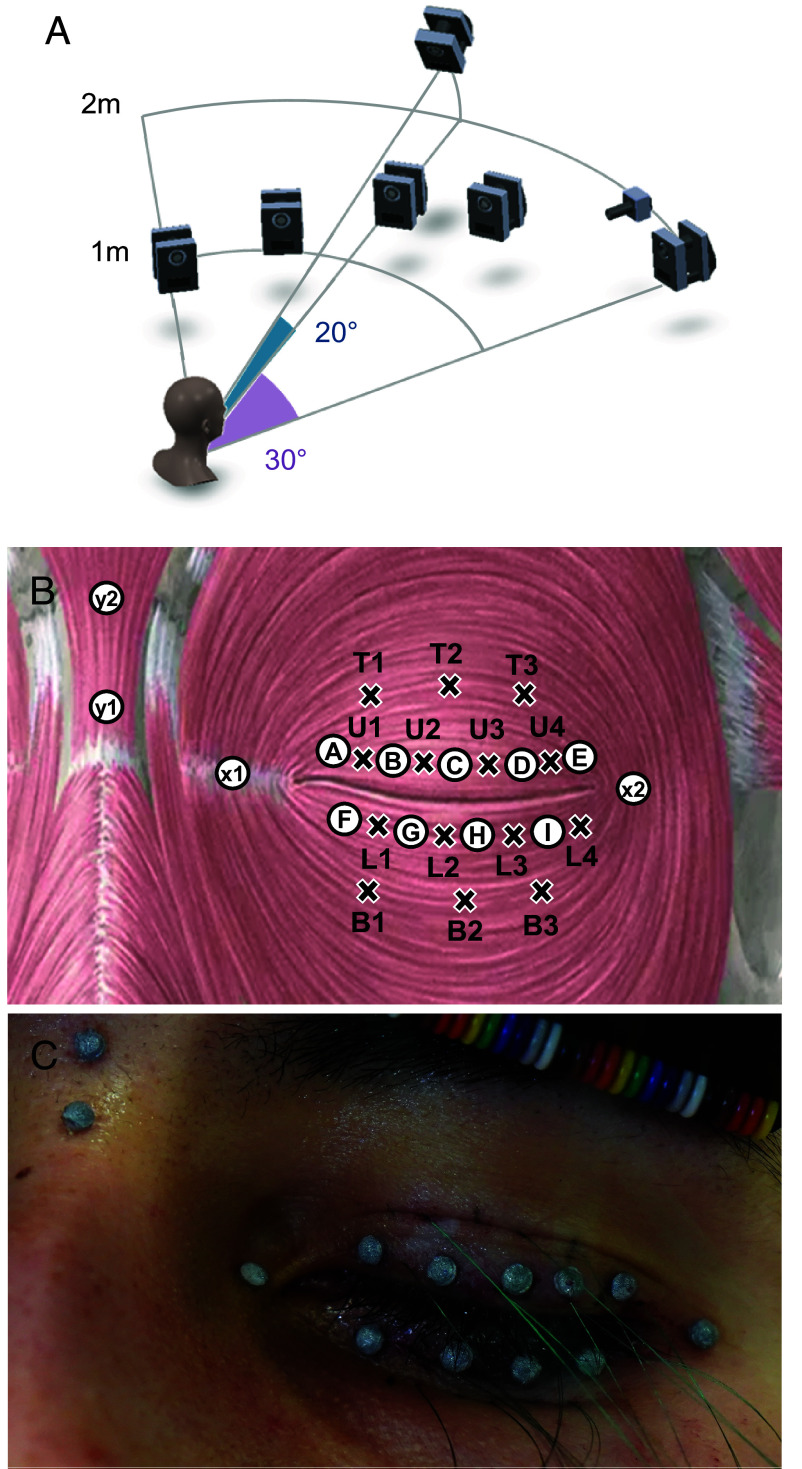
Experimental setup, and marker and electrode placement. (*A*) Approximate location and orientation of the motion-capture cameras relative to the participant’s head. (*B*) Target placement for the EMG electrodes (x) and the motion-capture markers (circle). Note that not all electrodes or markers were used for all participants (Table 1). (*C*) Representative photograph of the experimental setup. Fine wire electrodes (green) were placed first, followed by the motion capture markers (gray hemispheres).

**Fig. 2.**
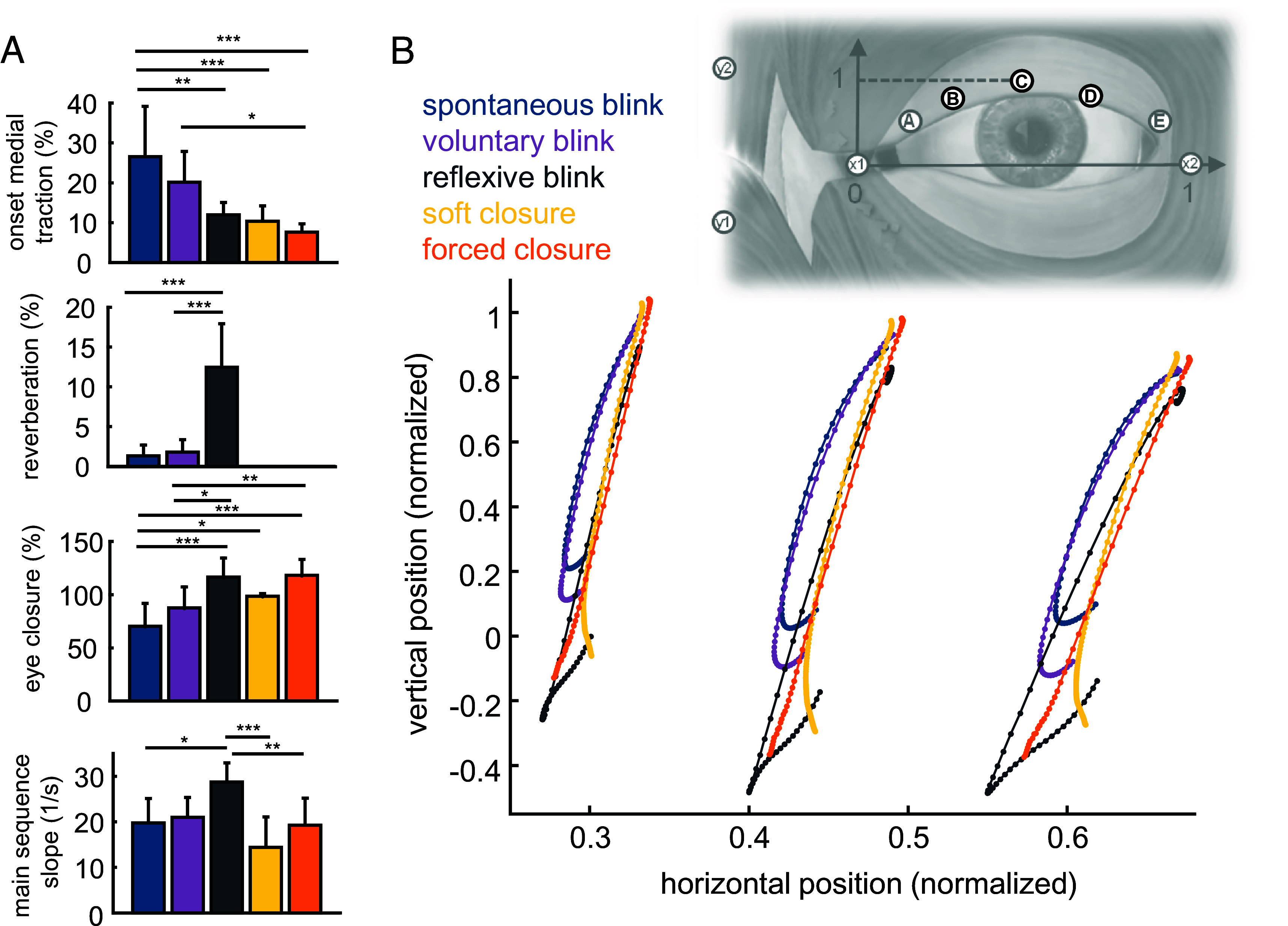
Upper eyelid kinematics during different eyelid behaviors. (*A*) Kinematic determinants of eyelid behavior. Bars show intersubject mean, and error bars show intersubject SD. *P < 0.05, **P < 0.01, ***P < 0.001. (*B*) Intersubject average kinematic trajectory of markers B, C, and D (left to right). All motions start at the upper free end of the trajectory. Plot origin is marker x1, located on the medial canthus. Decreasing horizontal position denotes motion in the medial direction (leftward on the plot). Each dot represents a motion capture frame; the time between dots is 2.5 ms. Individual subject trajectories are shown in *SI Appendix*, Fig. S2.

A set of five motion capture markers (2 mm hemisphere—adhesive back, Sisco Mocap LLC, Lake Elsinore, CA) along the eyelid margin (Fig. 1) was successfully tracked in three dimensions during five different eyelid behaviors. Four additional markers on the face were also tracked to establish a facial coordinate system. From the marker trajectory data, we identified four kinematic determinants that vary across eyelid behaviors (Fig. 2). The first of these, “onset medial traction,” describes medial motion of the eyelid early in the closure motion, and requires recording in at least two spatial dimensions to measure. “Reverberation” describes a sweeping overshoot of the upper eyelid beyond its complete (100%) closure position, and is also best described in at least two dimensions of motion. “Percent eyelid closure” denotes completeness of closure behavior. “Main sequence slope” denotes the slope of a first-order linear regression between closing phase amplitude and maximum vertical velocity (*SI Appendix*, Fig. S3). This relationship, which has been called “main sequence” in prior studies of eyelid movement (16, 18, 35), allows for velocity comparisons between different blink types by accounting for the effect of blink amplitude on maximum velocity. “Percent eyelid closure” and “main sequence slope” are easily reduced to one dimension in both measurement and analysis. In this study, we used three-dimensional motion capture measurements to construct a one-dimensional representation of these two quantities, allowing for comparison of our results with published one-dimensional studies.

The kinematic trajectories (Fig. 2*B*) show clear differences in the shape, speed, and amount of upper eyelid motion associated with each behavior. Spontaneous and voluntary blink deviate in the medial direction early in closure, and then return laterally. Reflexive blink moves further and faster than the other behaviors and exhibits a larger reverberation phase. Soft closure and forced closure follow similar trajectories to each other, but soft closure deviates laterally at the closed end of the motion.

Our kinematic determinant analysis (Fig. 2*A*), which quantifies these grossly observed differences, revealed that spontaneous blink is associated with significantly more onset medial traction (ANOVA with post hoc Tukey HSD, *P* < 0.002, Cohen’s d > 2.06 for all comparisons) and a lower percent closure (*P* < 0.016, Cohen’s d > 1.67) than any other behavior except voluntary blink. Reflexive blink had a moderate level of onset medial traction, but showed significantly more reverberation than any other behavior (*P* < 0.0001, Cohen’s d > 4.10) and a higher main sequence slope (*P* < 0.02, Cohen’s d > 1.67) than any other behavior except voluntary blink (*P* = 0.0501). Reflexive blink, soft closure, and forced closure produced full closure of the eyelid, whereas spontaneous blink produced significantly less than 100% closure (*P* = 0.016, Cohen’s d = 1.67). Although differences in eyelid motion between behaviors were subtle when viewed via high-speed video (*SI Appendix*, Fig. S1), motion capture revealed obvious kinematic differences that were remarkably consistent across people (*SI Appendix*, Fig. S2). Consistent kinematics that varied between behaviors were also observed in the markers on the lower eyelid margin (*SI Appendix*, Figs. S4 and S5).

### Muscle Activation and Marker-Based Excursion Dynamics Are Correlated.

Distributed intramuscular EMG was recorded from 14 bipolar fine-wire electrodes within one of each participant’s OO muscles (Fig. 1*C*), as well as two bipolar fine-wire electrodes in the contralateral central pretarsal OO muscle. Muscle activation was then calculated by filtering, rectifying, and integrating the EMG signal, followed by normalizing to the peak value of that electrode’s activation during maximum voluntary contraction (MVC) (forced closure). Activation onset time was calculated for each electrode for each event relative to the time when the first electrode showed a suprathreshold activation during that event.

Muscle excursion along the upper eyelid margin was calculated as the change in distance between each pair of motion capture markers, normalized to the resting distance between those same markers. Extrapolation of skin motion to muscle excursion in this way is uniquely possible in the eyelid, because the eyelid margin is connected along its length to the underlying orbicularis muscle (3), and eyelid skin is thinnest in the body with no subcutaneous fat layer in this position (8). Additionally, we used this excursion information to calculate shortening onset time, which describes the relative time at which shortening (negative excursion) first occurs in each muscle segment. Note that shortening is not expected to be an exact representation of muscle contraction or force production within a segment, as this relationship is not deterministic for muscle segments in series; because each segment can stretch all other segments in a “tug of war,” it is possible for a muscle segment to contract eccentrically. That said, muscle shortening *is* required for eyelid motion, such that shortening onset time provides useful information about when eyelid motion initiates within each segment. Shortening onset time was calculated for each segment for each event relative to the time of the first suprathreshold segment to shorten during that event; as such, shortening onset times are not directly comparable to activation onset times.

**Fig. 3.**
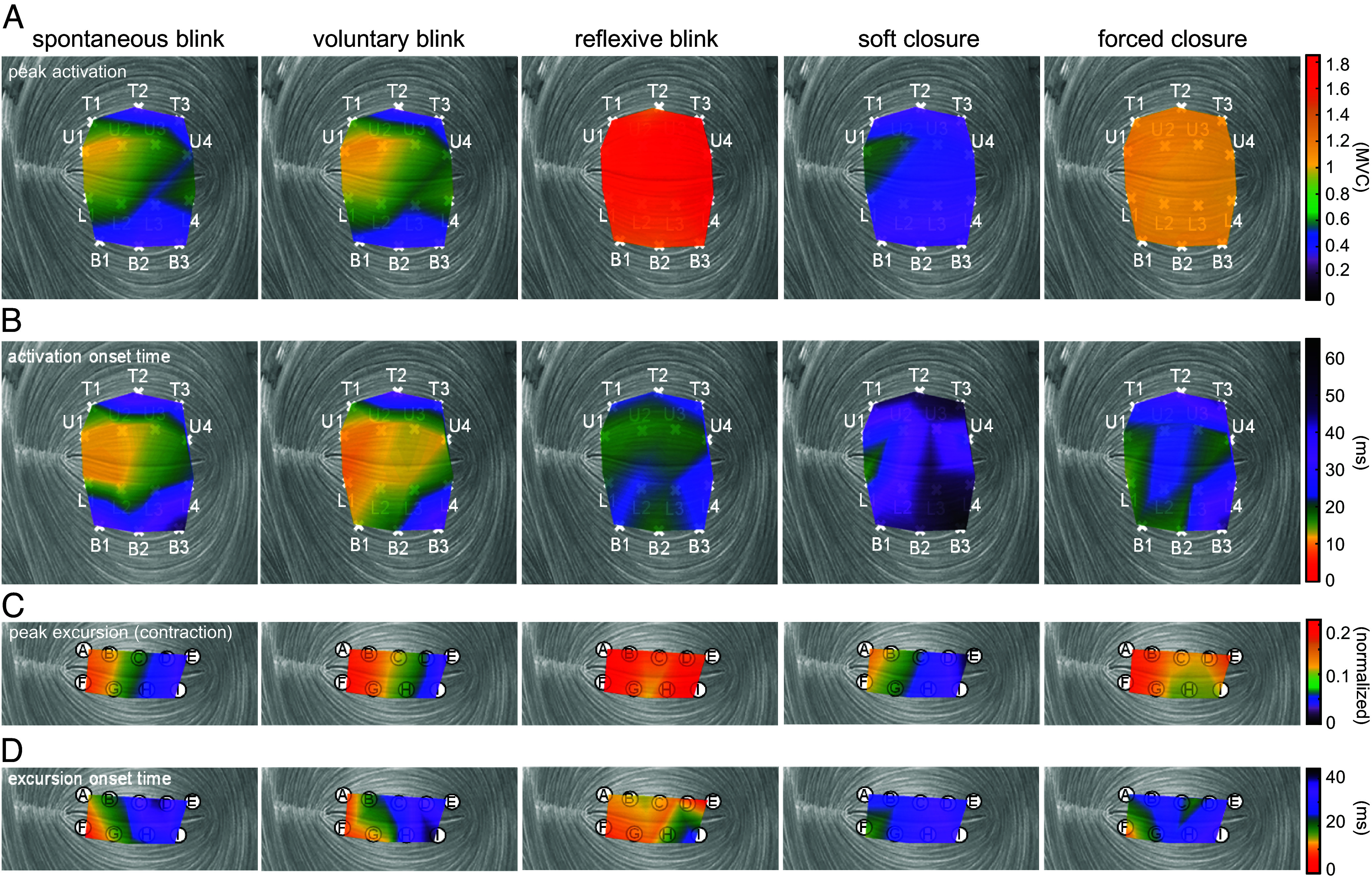
Temporospatial distribution of activation and excursion across the upper and lower eyelids. All values represent intersubject averages. Values are interpolated between discrete approximate recording locations. The left side of each plot is the medial side. (*A*) Peak activation, normalized to the peak value of EMG from that electrode during forced closure. (*B*) Activation onset time, calculated relative to the first activation onset across all electrodes, for each trial. (*C*) Peak negative excursion, which serves as a proxy for local muscle contraction. (*D*) Excursion onset time, calculated relative to the first excursion onset across all markers, for each trial.

Gross variation across the eyelid was observed in peak activation and activation onset time (Fig. 3 *A* and *B*), as well as peak muscle excursion and shortening onset time (Fig. 3 *C* and *D*). The most notable features were earlier and stronger muscle activation on the medial side for spontaneous and voluntary blink, versus near-simultaneous uniform activation across the eyelid in reflexive blink. Peak activation was linearly related to peak excursion (linear mixed effects model, *P* < 0.0001). Activation onset time was also linearly related to shortening onset time (*P* < 0.0001). Differences between behaviors are visible in slow-motion reconstructions of simultaneous muscle activation and eyelid kinematics (Movies S1–S6).

### Activation and Excursion Dynamics Vary Segmentally across the Eyelid.

**Fig. 4.**
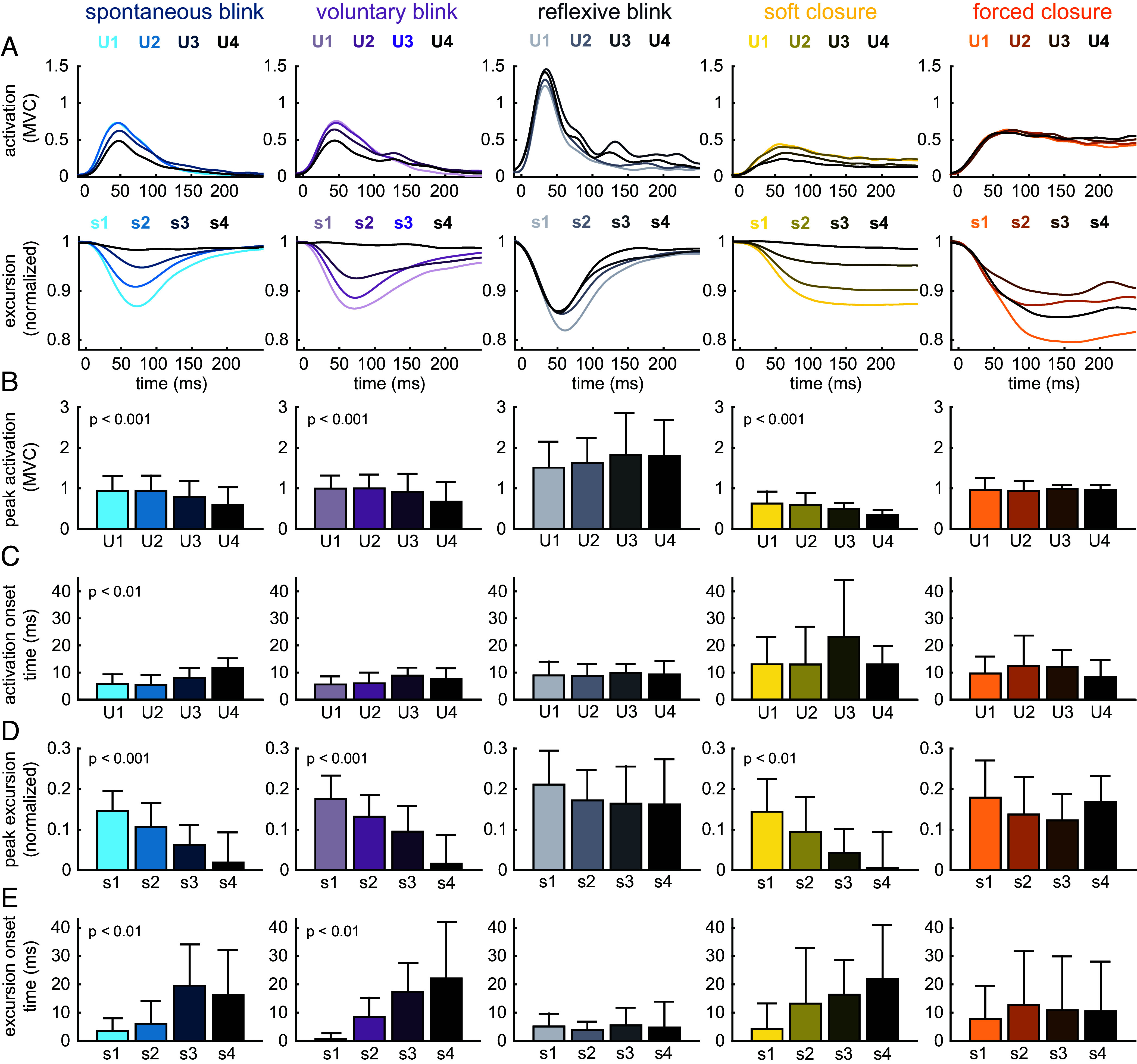
Segmental variation in activation and excursion across the upper pretarsal region of the OO muscle. Trajectories and bars show intersubject mean. Error bars show intersubject SD. U1-U4 are electrode sites (medial-to-lateral) and s1-s4 are excursions (changes in distance between markers, also medial to lateral). (*A*) Activation and excursion during each eyelid behavior. Activation values are normalized to the peak value of EMG from that electrode during forced closure. Excursion values are normalized to distance between markers with the eyelid open and at rest. Time t = 0 coincides with the first activation onset across all electrodes. (*B*) Peak activation in each electrode, normalized to the peak value of EMG from that electrode during forced closure. (*C*) Activation onset time, calculated relative to the first activation onset across the four electrodes in the upper pretarsal OO, for each trial. (*D*) Peak negative excursion, calculated based on intermarker distances. (*E*) Excursion onset time, calculated relative to the first negative excursion onset across the four intermarkers distances on the upper eyelid margin, for each trial.

Peak muscle activation (Fig. 4) varied significantly across the eyelid for spontaneous blink (ANOVA, *P* = 0.0004, η^2^ = 0.57), voluntary blink (*P* = 0.0002, η^2^ = 0.59), and soft closure (*P* = 0.001, η^2^ = 0.53), but not for reflexive blink (*P* = 0.58) or forced closure (*P* = 0.25). Peak excursion also varied significantly across the eyelid for spontaneous blink (*P* = 0.0007, η^2^ = 0.55), voluntary blink (*P* = 0.0002, η^2^ = 0.61), and soft closure (*P* = 0.008, η^2^ = 0.42), but not for reflexive blink (*P* = 0.65), or forced closure (*P* = 0.06). Shortening onset time varied significantly across the eyelid for spontaneous blink (*P* = 0.007, η^2^ = 0.43) and voluntary blink (*P* = 0.004, η^2^ = 0.46), but not for reflexive blink (*P* = 0.78), soft closure (*P* = 0.08), or forced closure (*P* = 0.31). However, during soft closure the average of the two medial segment (s1, s2) shortening onsets was significantly earlier (paired *t* test, *P* = 0.047, Cohen’s d = 0.95) than for the lateral segments (s3, s4). Activation onset time only varied significantly for spontaneous blink (*P* = 0.005, η^2^ = 0.45), but not for the remaining behaviors.

In the pretarsal region of the lower eyelid, no significant variation in peak muscle activation was found across the eyelid for any behavior (n = 6, ANOVA, 0.11 < *P* < 0.78 for the five behaviors). Onset timing varied significantly across the lower eyelid during spontaneous (*P* = 0.003, η^2^ = 0.60) and voluntary blink (*P* = 0.02, η^2^ = 0.54), but not for the remaining three behaviors (*SI Appendix*, Fig. S6). In the five subjects for whom we have excursion data from the lower eyelid margin, peak excursion varied significantly for spontaneous blink (*P* = 0.004, η^2^ = 0.75), voluntary blink (*P* = 0.005, η^2^ = 0.73), and soft closure (*P* = 0.03, η^2^ = 0.60), with the most excursion occurring on the medial side and the least on the lateral side. Excursion onset time in the lower eyelid also varied significantly for spontaneous blink (*P* = 0.009, η^2^ = 0.79), voluntary blink (*P* = 0.002, η^2^ = 0.87), and soft closure (*P* = 0.03, η^2^ = 0.71), but not for reflexive blink or forced closure. Activation recorded from the electrodes in the preseptal portion of the orbicularis showed similar medial-to-lateral patterns in these regions (*SI Appendix*, Fig. S7).

Additional electrodes were placed in the upper pretarsal region of the contralateral eyelid of five subjects, to permit analysis of side-to-side differences in muscle activation. Prior studies have shown that side-to-side difference in excitability of motor neurons can cause asymmetry of eyelid *kinematics* during voluntary and reflexive blink (36). Subjects were split into two groups—left or right asymmetric—by comparing activation onset time during voluntary blink (*SI Appendix*, Fig. S8*B*). Earlier average absolute onset time for a given side was assumed to correspond to greater excitability on that side. Side-to-side differences in activation onset time and peak activation were then calculated by subtracting the value of each metric on the low excitability side from that on the high excitability side (*SI Appendix*, Fig. S8*C*). There were no meaningful side-to-side differences in either metric, except for activation onset time at U1 during voluntary blink (*P* = 0.04, Cohen’s d = 1.33), which we note was the same metric used for grouping subjects based on excitability. For reflexive blink only, we also compared activation onset time for the side to which the air puff stimulus was applied (ipsilateral side) versus the contralateral side (*SI Appendix*, Fig. S8*D*). The ipsilateral side showed a faster onset time than the contralateral side for both U1 and U4 (*P* < 0.05, Cohen’s d > 2.51).

### Behavior-Specific Kinematic Features Are Associated with Changes in Activation Pattern.

**Fig. 5.**
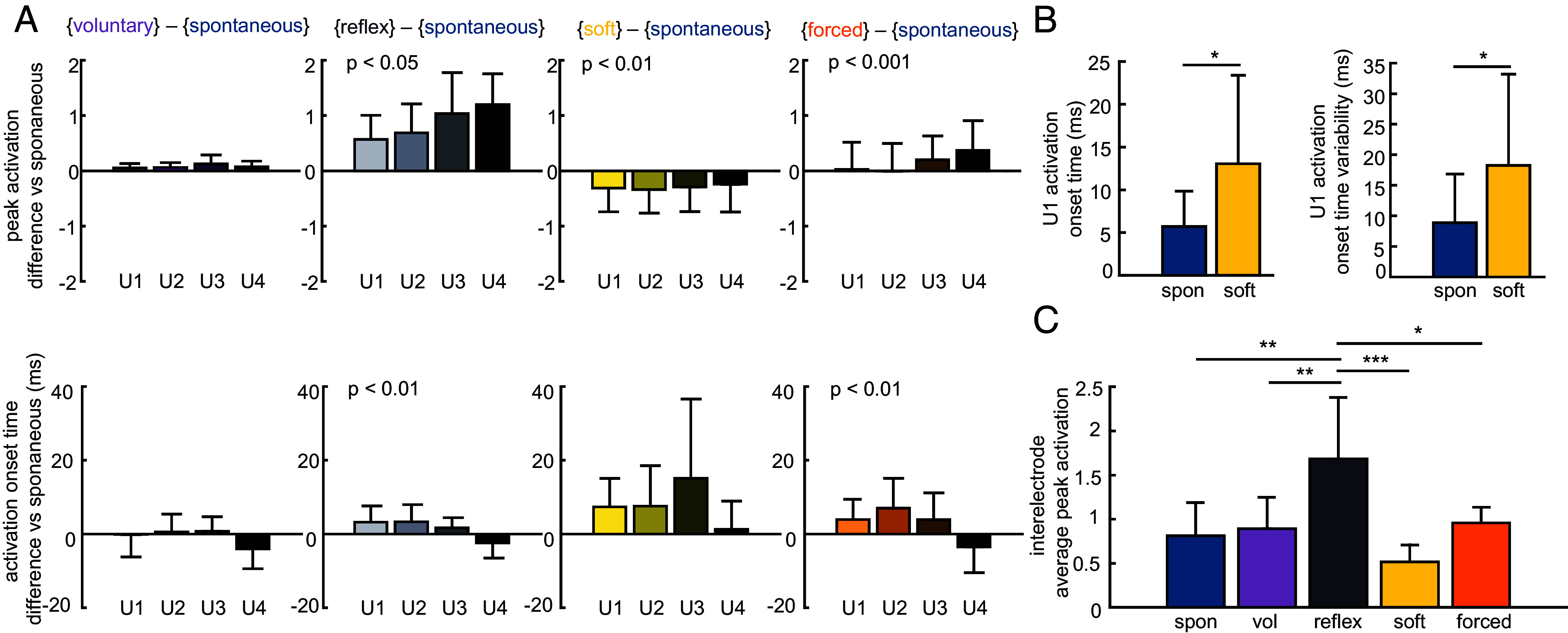
Differential segmental variation in activation magnitude and timing between behaviors. Bars show intersubject mean and error bars show intersubject SD. U1-U4 are electrode sites (medial-to-lateral). (*A*) Differences in peak activation and activation onset timing at each electrode between each spontaneous blink and each other eyelid behavior. Activation values are normalized to the peak value of EMG from that electrode during forced closure. Difference versus spontaneous that varies significantly as a function of electrode location is indicated by *P*-values in the top left corner (ANOVA). (*B*) Pairwise comparison of electrode U1 timing between spontaneous blink and soft closure. (*C*) Comparison of interelectrode average peak activation between all behaviors. Peak activation values are normalized to peak activation during forced closure. *P < 0.05, **P < 0.01, ***P < 0.001.

We hypothesized that the high level of “onset medial traction” observed during spontaneous blink could be explained by changes in the *pattern* of activation across the OO compared to the other behaviors. To evaluate this, we subtracted each electrode’s peak activation during spontaneous blink from its peak activation during all other behaviors (Fig. 5*A*) and then tested to see whether the resultant differences were significantly impacted by electrode number (ANOVA). This test shows significance only if the activation pattern is different between behaviors, and will not show significance for a change in magnitude of activation across the OO that maintains the same pattern between behaviors. We found significant effects of electrode number on peak activation differences for reflexive blink (ANOVA, *P* = 0.02, η^2^ = 0.38), soft closure (*P* = 0.006, η^2^ = 0.44), and forced closure (*P* = 0.0003, η^2^ = 0.59) versus spontaneous blink, but not for voluntary blink (*P* = 0.87). We repeated the analysis for activation onset time, and found significantly different patterns of activation timing for reflexive blink (*P* = 0.003, η^2^ = 0.48) and forced closure (*P* = 0.008, η^2^ = 0.42) versus spontaneous blink. We did not see significant effects between electrodes for either voluntary blink (*P* = 0.15) or soft closure (*P* = 0.12) versus spontaneous blink, indicating that the kinematic differences between spontaneous blink and soft closure may not be driven by significant variations in activation timing.

In light of these findings, we further hypothesized that the kinematic differences between soft closure and spontaneous blink could also be explained by changes to the timing of activation onset explicitly on the medial side (specifically electrode u1). In a pairwise comparison, we found that the activation onset time at electrode u1 was significantly earlier during spontaneous blink than during soft closure (Fig. 5*B*, paired *t* test, *P* = 0.03, Cohen’s d = 0.95). We also found a significant difference in the intrasubject *variability* of onset time at electrode u1—defined as each subject’s across-trial SD of onset time for that electrode—between spontaneous blink and soft closure (*P* = 0.02, Cohen’s d = 1.05).

We hypothesized that the primary kinematic characteristics that distinguish reflexive blink from the other four behaviors (i.e., reverberation and main sequence slope) could be explained by an increase in peak activation across the whole eyelid. Indeed, we found that average peak activation was significantly higher for reflexive blink than for spontaneous blink (repeated measures ANOVA with post hoc Tukey HSD, *P* = 0.0006, Cohen’s d = 2.26), voluntary blink (*P* = 0.002, Cohen’s d = 2.09), soft closure (*P* < 0.0001, Cohen’s d = 2.99), and forced closure (*P* = 0.01, Cohen’s d = 1.70). We also note that there was no significant effect of electrode location on activation onset time for reflexive blink (*P* = 0.97), that the average relative onset time across the eye was low (9.2 ms), and that the intrasubject variability for timing of reflexive blink was small, comparable to that of spontaneous blink (11.1 ms for reflexive versus 8.6 ms for spontaneous). Together, these imply that reflexive blink was characterized by muscle segments consistently, simultaneously activating to a high level.

## Discussion

Our results show that each behavior is characterized by unique patterns of OO activation and resultant eyelid kinematics specific to that behavior’s functional purpose. Two-dimensional evaluation of eyelid kinematics revealed critical differences in eyelid motion between behaviors (Fig. 2*A*), which were consistent across people. Our 1D kinematics results (main sequence slope and eye closure) also varied between behaviors in ways that mirrored what has been reported in other studies (15, 16, 37, 38). We found significant variation in muscle activation and excursion patterns across the eyelid (Figs. 3 and 4), which cannot reasonably be explained by a conventional single-segment model of the OO. These patterns (both intensity and timing) also varied in different ways during different behaviors (Figs. 4 and 5). Perhaps the most likely explanation for these behavioral variations in activation pattern is that the segments of the OO are composed of alpha motoneurons of different sizes (39), and the sequence of activation corresponds to size-ordered recruitment of fibers. Specifically, if the medial OO contains a relatively higher concentration of small motoneurons, then a slowly developing neural drive would first activate the medial OO. We note that this theory permits a decoupling of peak activation from the speed at which that activation develops; the latter is more closely tied to activation onset time. Herein we discuss the specific kinematic features we observed for each behavior, postulate how those features relate to function, and link those features to specific activation patterns in the OO.

Spontaneous blink was characterized by early lateral-to-medial motion of the eyelid, with a small amount of reverberation and incomplete closure (Fig. 2*A*). This elevated onset medial traction is driven by earlier, stronger activation and contraction in the medial OO segments than the lateral segments. On the whole, the unique motion path of the eyelid during spontaneous blink appears to be tied to both timing and intensity of segmental activation. Soft closure was found to have a different pattern of activation intensity across the eyelid compared to spontaneous blink (Fig. 5*A*), but there were no significant differences in activation timing (due in part to high variance between subjects in onset timing during soft closure). However, a closer look at the most medial electrode for the two behaviors showed that the medial OO activates more consistently earlier in spontaneous blink than soft closure (Fig. 5*B*). We posit that this early medial activation in spontaneous blink may serve to locally reorient the fibers of the OO in the medial direction, such that subsequent activation of the lateral segments results in a trajectory that skews more medial than the other behaviors (Fig. 2*B* and *SI Appendix*, Fig. S2). The resultant early lateral-to-medial motion may be related to drawing tear film across the eye and pushing excess tears into the outflow ducts in the lower-medial aspect of the eye. Based on the muscle activation and excursion patterns late in the behavior, the small amount of reverberation at the end of spontaneous blink appears likely to be caused by elastic return of the eyelid upon relaxation of the medial OO, rather than active repositioning of the eyelid in the lateral direction. Voluntary blink was similar to spontaneous blink, with no significant differences in kinematics, muscle activation, or muscle excursion. This provides strong evidence that humans are able to reproduce volitionally the activation patterns that drive involuntary spontaneous blink, resulting in functionally equivalent eyelid motion.

Reflexive blink was characterized by a large amount of reverberation, higher contraction velocities, and more complete closure than the other behaviors (Fig. 2*A*). This is consistent with the goal of reflexive blink, which is to protect the eye as quickly and completely as possible from external insult. As the upper lid closes quickly to meet the lower lid, it overshoots its target and then “bounces back” to a more lateral endpoint, which is in a similar location to the endpoint for soft closure. This rapid motion is driven by a near-simultaneous (within 10 ms) activation of the entire pretarsal OO, with a higher peak activation than observed in any other behavior (Fig. 5*C*). This intense activation was not sustained for a long period of time, diminishing to a fraction of MVC within 100 ms. Reflexive blink, which is an involuntary behavior, was faster and involved more activation than volitionally closing the eyes “as hard as possible” (forced closure).

In soft closure, the eyelid consistently closed more fully than in spontaneous blink (Fig. 2*A*), but was driven by slightly lower peak activation (Figs. 4*B* and 5*A*). This may be linked to more sustained activation: Whereas activation for blink peaks quickly and then diminishes, activation for soft closure is much more gradual. Complete eye coverage is the dominant feature of soft closure, to block light and ensure wetting and protection of the eye. Peak activation and excursion both varied significantly across the eyelid during soft closure, and a medial bias was observed in both signals. Although both voluntary blink and soft closure involve volitional closure of the eyelid, we did not observe in soft closure the onset medial traction that was characteristic of spontaneous and voluntary blink. We also saw the largest average intrasubject (within subject, between trial) variation in activation onset timing for soft closure, implying that subjects do not always activate the OO in the same pattern when gently closing their eyes. One explanation for these differences between soft closure and voluntary blink is that soft closure may involve a complex interaction between the OO and the LPS, from which we did not record activation. The kinematics of forced closure were similar to those of soft closure. Relative activation intensity across the eyelid could not easily be parsed in isolation for forced closure, because forced closure served as the MVC baseline to which all behaviors were normalized. However, the pattern of peak activation and onset timing during forced closure were both significantly different from spontaneous blink (Fig. 5*A*).

Lower-eyelid kinematics differed between behaviors, in ways that were consistent across subjects (*SI Appendix*, Figs. S4 and S5). Activation in the lower pretarsal and the upper and lower preseptal regions showed similar medial-to-lateral patterns to those observed in the upper pretarsal region, implying that there may be shared innervation between these regions (*SI Appendix*, Fig. S7). Although no significant differences were observed in activation timing between sides of the face, the onsets were not precisely simultaneous (*SI Appendix*, Fig. S8). While this could be due to slight asymmetries in muscle activation across the face, it also may be the result of differences in medial-to-lateral placement of the electrodes within each eyelid. Although every effort was made to place corresponding electrodes in identical locations on each side of the face, our results show high sensitivity of timing to medial-lateral location within the OO.

Our study was run on young, healthy participants with characteristic “double eyelid” morphology in an upright seated position; further studies would be necessary to understand the impact of age, pathology, eyelid configuration, and posture on eyelid neuromechanics. Based on published literature, we expect that race [especially eye shape (40)] and age (11, 41) are likely to show meaningful differences in eyelid mechanics. It is also possible that the OO activation patterns necessary to produce functional blink may differ when the head is not upright, due to the nontrivial effect of gravity on the eyelid. We also note that we did not record EMG from the LPS due to concern for the safety of the eye. Differential activation of the upper LPS, which retracts the eyelid, may contribute to the observed differences in kinematics across behaviors (especially in soft closure, as described above).

These results have significant implications for the development of neuroprosthetic systems to restore natural blink in persons with facial paresis. Such systems seek to decode motion from EMG recordings of the sound side OO, and “replay” those motions by stimulating the paralyzed OO (42–46). Based on the complexities we observed in OO activation between behaviors, decoding eyelid motion from EMG signals may require an array of electrodes spread across at least the pretarsal region of the OO. Perhaps more importantly, due to reverse order recruitment during artificial stimulation (47, 48), neuroprosthetic recreation of these motions will likely require precise segmental recruitment of OO fibers, with independent control of activation timing and intensity. Current “all-at-once” stimulation approaches (24, 49, 50) are likely to create motions that mimic the protective functions of reflexive blink, rather than the natural wetting and tear-clearing functions of spontaneous blink. These results of this work may also inform future mechanistic models of eyelid function, and lead to enhanced diagnosis and treatment of eyelid pathology.

## Methods

### Participants.

All experiments involving human subjects were conducted under the approval of the Institutional Review Board of the University of California, Los Angeles. All participants were informed of the risks and potential side effects of participation, and provided written consent before the beginning of experiments. Eight subjects [five female and three male; mean age 27.3 (22 to 33) y] participated in the experiment (full demographic information in Table 1). Of note, all subjects, including those of Asian descent, had double eyelid morphology.

**Table 1.** Participant demographics and marker/electrode set for each subject

Subject	Age (years)	Sex	Race	Eyelid type	Eyelid side	Motion capture	EMG electrodes
**1**	33	Male	White	Double	Right	Upper	U1 U2 U3 U4 T2 L1 L3 B2
**2**	25	Female	Asian	Double	Right	Upper	U1 U2 U3 U4 T2 L1 L3 B2
**3**	26	Female	White	Double	Left	Upper Lower	U1 U2 U3 U4 T1 T2 T3 L1 L2 L3 L4 B1 B2 B3
**4**	26	Female	Asian	Double	Right	Upper Lower	U1 U2 U3 U4 T1 T2 T3 L1 L2 L3 L4 B1 B2 B3
**5**	27	Female	White	Double	Right	Upper Lower	U1 U2 U3 U4 T1 T2 T3 L1 L2 L3 L4 B1 B2 B3
**6**	30	Male	White	Double	Left	Upper	U1 U2 U3 U4 T1 T2 L1 L2 L3 L4 B1 B2
**7**	22	Female	Black	Double	Left	Upper Lower	U1 U2 U3 U4 T1 T2 T3 L1 L2 L3 L4
**8**	29	Male	Asian	Double	Left	Upper Lower	U1 U2 U3 U4 T1 T2 T3 L1 L2 L3 L4 B1 B2 B3

### Experimental Setup.

Prior to participating in experiments, participants removed makeup and any jewelry from their faces. For each participant, either the right or left eyelid was selected at random (physical coin flip) for placement of the electrodes and markers. Topical anesthetic (Lidocaine Prilocaine 20%, Phenylephrine 0.25%) was applied to the skin on the eyelid 30 min prior to placement of the electrodes. This combination is commonly used in the clinical setting to prepare the eyelid for injections and is not known to have any neuromuscular effects. After 30 min, topical anesthetic was removed with 70% isopropyl alcohol pads, and target locations for needle insertion were marked on the eyelid using a surgical marking pen. With the subject lying supine on the patient table, 14 bipolar fine-wire electrode pairs (eight pairs on pretarsal OO and six pairs on preseptal OO) were then placed in the OO via 27-gauge needles by an ophthalmic plastic and reconstructive surgeon (Fig. 1). An electrical ground was set using a surface EMG electrode attached over the clavicle. In six subjects, two additional bipolar pairs were placed at locations u1 and u4 on the contralateral eyelid, to allow for symmetry measurements. Each target needle insertion point was marked approximately 3 mm lateral to the desired EMG collection point, leaving space for the hook at the end of wire to lie flat in the muscle in the plane of the eyelid. The free end of each wire was connected to custom connectors made from music wire spring (51) (EI 008A 07 M, Lee Spring, Brooklyn NY). These connectors were affixed to the head and held hanging in front of the subject’s forehead by a headband. EMG signals were routed from the fine-wire electrodes through this electrode system to a digital amplifier and signal processor (RZ5D-Base-Processor, Tucker-Davis Technologies Inc., Alachua, FL). After placement of each electrode pair, the EMG signal from that electrode pair was inspected in real time for signal-to-noise ratio and motion robustness. All EMG data were sampled at 6103.5 Hz, with the exception of subject 1 (sampled at 1017.3 Hz).

After all electrodes were inserted, 13 reflective markers (2 mm Hemisphere—adhesive back, Sisco Mocap LLC, Lake Elsinore, CA) were placed along the upper and lower eyelid margins between the electrode sites (Fig. 1 and Table 1) using a dissolvable skin adhesive. One additional marker was placed on the upper eyelid relative to the lower eyelid because the upper eyelid is longer. The subject was then helped slowly to a seated position, facing an array of six motion capture cameras (Vantage, Vicon Motion Systems, Oxford UK) and 1 high-definition video camera (Vue, Vicon Motion Systems). Reflective marker trajectories were recorded in three dimensions (3D) at 400 frames per second. High-definition video was recorded at 120 frames per second. Motion capture data were time-synced with EMG data via a digital hard sync. After completion of the experiment, electrodes were carefully removed and markers were detached by the clinician.

### Eyelid Behaviors and Experimental Protocol.

While EMG and motion capture data were recorded, each subject performed five different eyelid behaviors: spontaneous blink, voluntary blink, reflexive blink, soft closure, and forced closure. Between twenty and forty discrete samples were collected for each behavior (e.g., 40 consecutive spontaneous blinks). Protocols for each blink type were as follows:

1. Spontaneous blink: Participants watched a video clip on a small screen far from their face, to minimize tracking saccades. One researcher stood beside the screen and silently counted discrete blinks. The first five blinks, during which the subjects might be overly aware of their blink motions, were discarded. The video clips were chosen to avoid reactionary facial expressions (e.g., not funny or sad), but interesting enough for participants to concentrate and become unaware of their blink motions.
2. Voluntary blink: Subjects were instructed to fixate on a single point in the distance with the eyes in primary position and the plane of the face perpendicular to the floor. A digital tone was played every 2 s. Subjects were instructed to blink each time they heard the tone.
3. Reflexive blink: Subjects were instructed to fixate on a single point in the distance with the eyes in primary position and the plane of the face perpendicular to the floor. From a position outside of the subject’s visual field, a researcher directed an air puff toward the eyes at inconsistent time intervals, leading to reflexive closure.
4. Soft closure: Subjects were instructed to fixate on a single point in the distance with the eyes in primary position and the plane of the face perpendicular to the floor. A digital tone was played at alternating 5 and 2 s intervals. Subjects were instructed to close their eyelids softly and hold for 5 s and then open for the following 2 s. Subjects were allowed to blink during the “open” phase, but trials were discarded if they blinked immediately before initiating soft closure.
5. Forced closure: Subjects were instructed to fixate on a single point in the distance with the eyes in primary position and the plane of the face perpendicular to the floor. A digital tone was played at alternating 5 and 2 s intervals. Subjects were instructed to close their eyelids as hard as they could and hold for 5 s, and then open for the following 2 s. Subjects were allowed to blink during the open phase, but trials were discarded if they blinked immediately before initiating forced closure.

The order of behaviors tested was randomized for each subject to minimize order bias. Subjects rested for at least 2 min between blink types to minimize muscle fatigue.

### Electromyography Data Processing and Analysis.

EMG data were notch filtered at 60 Hz and its integer multiples up to 360 Hz to eliminate ambient electrical noise. The data were then low-pass filtered at 400 Hz using a zero-phase Butterworth filter (“filtfilt,” MATLAB R2023b, Mathworks Inc, Natick, MA). To obtain the signal envelope, the filtered signals were rectified and zero-phase low-pass filtered at 30 Hz. All EMG signals were normalized to the average maximum value of the EMG envelope across forced closure trials, which was used as a proxy for MVC, on a per-subject basis. The activation trajectories in Fig. 4 represent intersubject average normalized EMG envelope for each electrode.

From the normalized EMG envelope, we extracted several key features for each blink event. Reported values for each EMG feature (bar graphs in Figs. 4 and 5 and *SI Appendix*, Figs. S6 and S7) are averaged across trials and then averaged again across subjects for each behavior, and intersubject SD is reported. Peak activation was calculated as the maximum value of the normalized EMG envelope during each blink event. Activation onset time was calculated using a two-threshold rising edge detection method. Specifically, the time derivative of the normalized EMG envelope was compared to two threshold lines. The lower of these thresholds was set for all subjects and behaviors at 2 s^−1^. The higher threshold value, which served as a safeguard against false-positive EMG onsets, was set by multiplying the average normalized EMG envelope value during the initial at-rest phase by a constant integer value between 2 and 4. This integer multiplier was determined for each behavior for each subject as the minimum value that consistently eliminated false-positive event identification. For each blink event, the instant of activation onset was identified as the time point when the derivative of the normalized EMG envelope crossed the lower threshold, and then cross-checked to ensure that it then crossed the higher threshold within 0.2 s. Activation onset times are reported relative to the time of the first suprathreshold electrode for a given event; onset time for each electrode was calculated by subtracting this “fastest” onset time from all other onset times. Note that the heatmaps in Fig. 3 show the relative onset time between all 14 electrodes, whereas the bar graphs in all other figures show the relative onset only between the relevant electrode subset (e.g., Fig. 4 shows relative onset times between electrodes u1-u4).

### Motion Capture Data Processing and Analysis.

Typical eyelid closure events were approximately 100 ms in duration, such that a capture rate of 400 frames per second provided approximately 40 samples for each trial. Gaps in marker tracking smaller than 10 samples (25 ms) were filled via linear interpolation. Gaps larger than 10 samples were excluded from analysis. After gap filling, data were upsampled by 10 times via linear interpolation, and then zero-lag lowpass filtered at 20 Hz. To eliminate translation and rotation of the head from our measurements, marker positions were transformed to a coordinate frame grounded to the face. The origin of this coordinate frame was the marker x1 (placed on the medial canthus), the y-axis was the vector from y1 to y2, the x-axis was the orthogonal projection of x2 onto the y-axis, and the z-axis was the right-handed orthogonal to the x and y axes. To allow for comparisons across subjects with different eyelid shapes and sizes, marker positions in the x-axis (medial-lateral) direction were normalized to the width of the eye, defined as the x-axis distance between markers x1 and x2 with the eyelid open and at rest. Marker positions in the y-axis were normalized to the height of the eye, defined as the y-axis distance between markers C and x1 with the eyelid open and at rest. Kinematic trajectories are shown in the x-y plane, from the eye-open state to 50 ms past the lowest point in vertical position. Four kinematic determinants were defined to compare upper-eyelid kinematics between different eyelid behaviors:

1. Onset medial traction (%) is a measure of how much each marker moves in the medial direction at the initiation of the eyelid behavior. It is calculated as the distance traveled in the x-direction during the first 10 percent of the total event time, as a percent of the distance traveled in the x-direction at maximum closure.
2. Reverberation (%) is a measure of how much a marker bounces back after reaching the lowest point in its trajectory. We observed empirically that in some trials, the end of this reverberation phase was characterized by an inflection in the spatial trajectory of the markers (mathematically, a change in sign of d^2^y/dx^2^). In other cases, the end of reverberation was characterized by the marker looping back on its path, such that there was an intersection point in its trajectory. Reverberation was calculated as the difference in y-coordinates between the lowest y-axis value in the trajectory and either the inflection point or the intersection point (whichever distance was larger), as a percentage of the height of the eye. In cases where no inflection or intersection point was observed, reverberation was set to a value of 0.
3. Eye closure (%) is calculated as the difference in each marker’s y-coordinates between the eye-open state and lowest y-axis value in the trajectory, as a percentage of the same distance as calculated during soft closure. This assumes that soft closure represents complete closure of the upper eyelid.
4. Main sequence slope (s^−1^) is calculated as the slope of a first-order linear regression between the maximum vertical velocity of a marker during a single closing event (e.g., one blink) and the amplitude of that marker’s vertical movement during the closing phase of that closing event. For each subject for each behavior, the linear regression was calculated using individual data from all markers (A-E) from all trials (*SI Appendix*, Fig. S3).

All kinematic determinants except for main sequence slope were calculated for each marker (A-E), and then averaged between those markers for each trial. In addition to these planar kinematic determinants, we also calculated the distance between markers as a real-time proxy for muscle excursion. This is uniquely possible at the eyelid margin because the skin at the margin is adhered to the OO muscle along its length. Muscle excursion was calculated as the difference between the instantaneous 3D distance between markers and the 3D distance between those same markers at rest in the eye-open state, normalized to the distance between the markers in the eye-open state. Excursions s1, s2, s3, and s4 represent the change in distance between the markers A-B, B-C, C-D, and D-E respectively. Excursions i1, i2, and i3 represent the change in distance between F-G, G-H, and H-I respectively. Peak excursion was defined for each marker pair for each blink event as the absolute value of the most-negative value of muscle excursion between that pair. Excursion onset was identified similarly to activation onset, except the thresholds were applied to excursion and not to its derivatives. The lower threshold for excursion onset time was 0.01. Excursion onset time was calculated by subtracting the fastest excursion onset time from all onset times. Note that the heatmaps in Fig. 3 show the relative excursion onset time between all seven muscle excursions (s1-s4 and i1-i3), whereas the bar graphs in all other figures show the relative onset only between the relevant marker subset (e.g., Fig. 4 shows relative onset times for distances s1 to s4).

### Statistical Analysis.

Statistical analyses were conducted in MATLAB R2023b (Mathworks Inc, Natick, MA). All bar graphs represent intersubject mean, and error bars show intersubject SD. For the heatmaps (Fig. 3), linear two-dimensional interpolation was used in the space between electrode/marker locations; no extrapolation was performed. Data were checked for normality using a Kolmogorov–Smirnov test prior to any parametric statistical tests. Kinematic differences across blink types were evaluated for significance via a one-way ANOVA. In cases where the ANOVA showed a significant effect of blink type, a post hoc Tukey HSD was used for pairwise comparisons. Significant results are reported with both *P*-value and effect size (Cohen’s d). Repeated measures ANOVA was used to evaluate the effect of electrode position (u1 to u4) or excursion location (s1 to s4) on a given measure (e.g., peak activation). Significant results are reported with both *P*-value and a measure of effect size (η^2^). Hypotheses involving single pairwise comparisons (Fig. 5*B*) and one-sample comparison (*SI Appendix*, Fig. S8) were tested using a two-sided pairwise *t* test and one-sided *t* test, respectively. Significant results were reported with both a *P*-value and effect size (Cohen’s d). Correlations were evaluated using a first-order linear mixed effects model with subject as the random effect. All statistical tests were performed at a significance level of α = 0.05.

## Supplementary Material

Appendix 01 (PDF)

Movie S1. Spontaneous blink – dynamic muscle activation patterns and eyelid kinematics.

Movie S2. Voluntary blink – dynamic muscle activation patterns and eyelid kinematics.

Movie S3. Reflexive blink – dynamic muscle activation patterns and eyelid kinematics.

Movie S4. Soft closure – dynamic muscle activation patterns and eyelid kinematics.

Movie S5. Forced closure – dynamic muscle activation patterns and eyelid kinematics.

Movie S6. Side-by-side comparison of activation and kinematics between spontaneous blink, reflexive blink, and soft closure.

## Data Availability

EMG and motion capture data collected from experiments have been deposited to FaceBase and are available under Record ID 88-28BJ (10.25550/88-28BJ) (52). All other data are included in the article and/or supporting information.
