# Supplementary material for: Human eyelid behavior is driven by segmental neural control of the orbicularis oculi
Source: Proc Natl Acad Sci U S A. 2025 Aug 7;122(32):e2508058122. doi: 10.1073/pnas.2508058122 (PMC12358864; doi:10.1073/pnas.2508058122)
Supplement: Supplementary file 1 — Appendix 01 (PDF) [file pnas.2508058122.sapp.pdf]

SUPPORTING INFORMATION

Supporting Videos

Video S1: Spontaneous blink – dynamic muscle activation patterns and eyelid kinematics.

Video S2: Voluntary blink – dynamic muscle activation patterns and eyelid kinematics.

Video S3: Reflexive blink – dynamic muscle activation patterns and eyelid kinematics.

Video S4: Soft closure – dynamic muscle activation patterns and eyelid kinematics.

Video S5: Forced closure – dynamic muscle activation patterns and eyelid kinematics.

Video S6: Side-by-side comparison of activation and kinematics between spontaneous blink, reflexive blink, and soft closure.

Supporting Figures

Figure S1

Figure S2

Figure S3

Figure S4

Figure S5

Figure S6

Figure S7

Figure S8

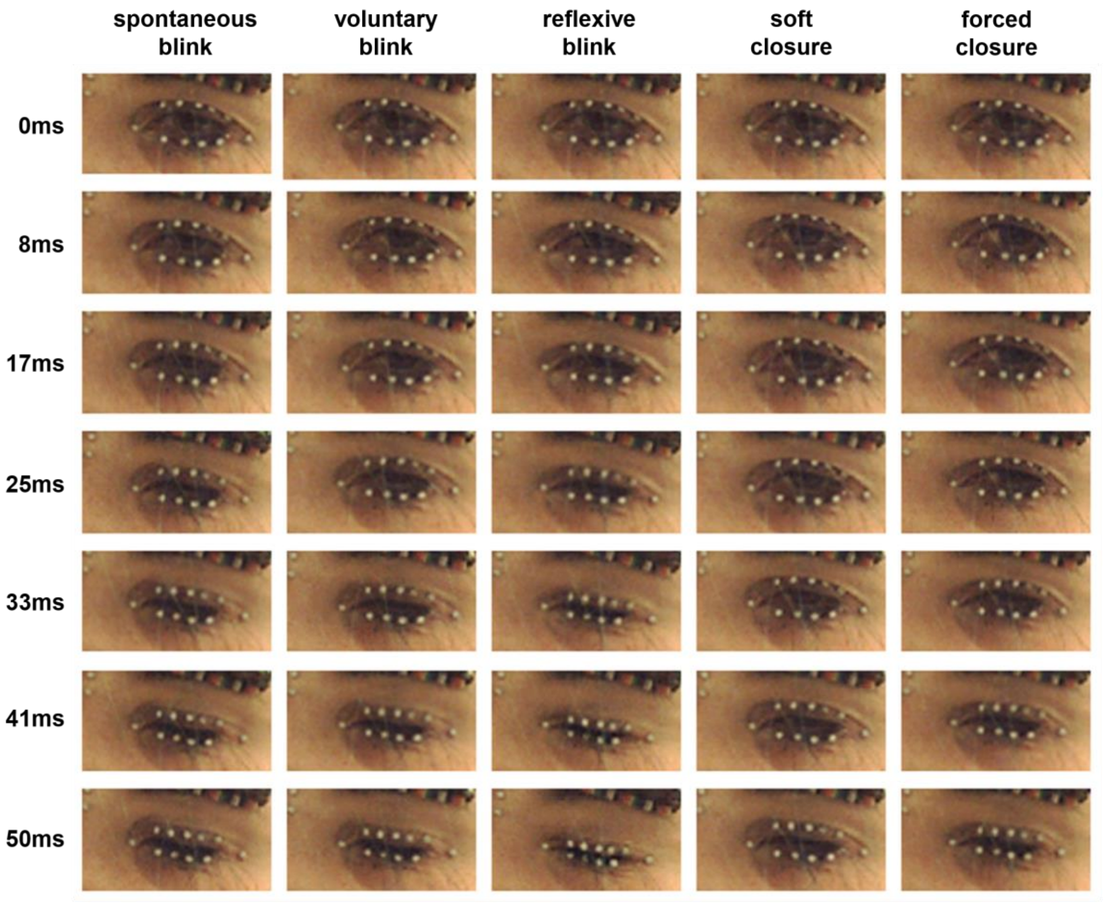

Figure S1. Time series of eyelid motion during different eyelid behaviors, extracted from video frames.

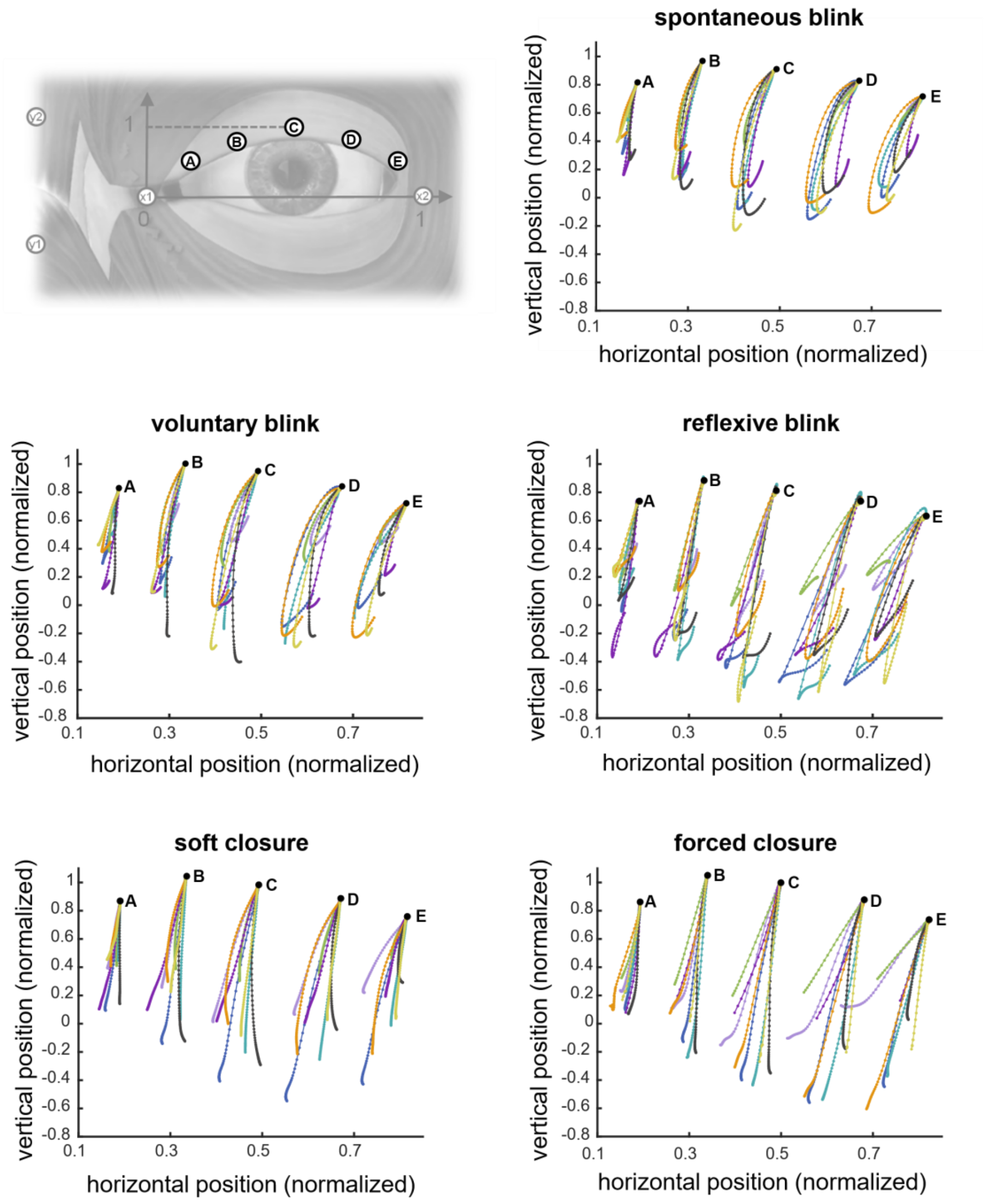

Figure S2. Individual subject upper eyelid kinematics during different eyelid behaviors. Lines represent intrasubject average kinematic trajectories. All motions start at the black dot; to facilitate comparison all trajectories are uniformly translated so that their starting point coincides with the inter-subject average starting point. Each colored dot represents a single motion capture frame; the time between dots is 2.5 ms.

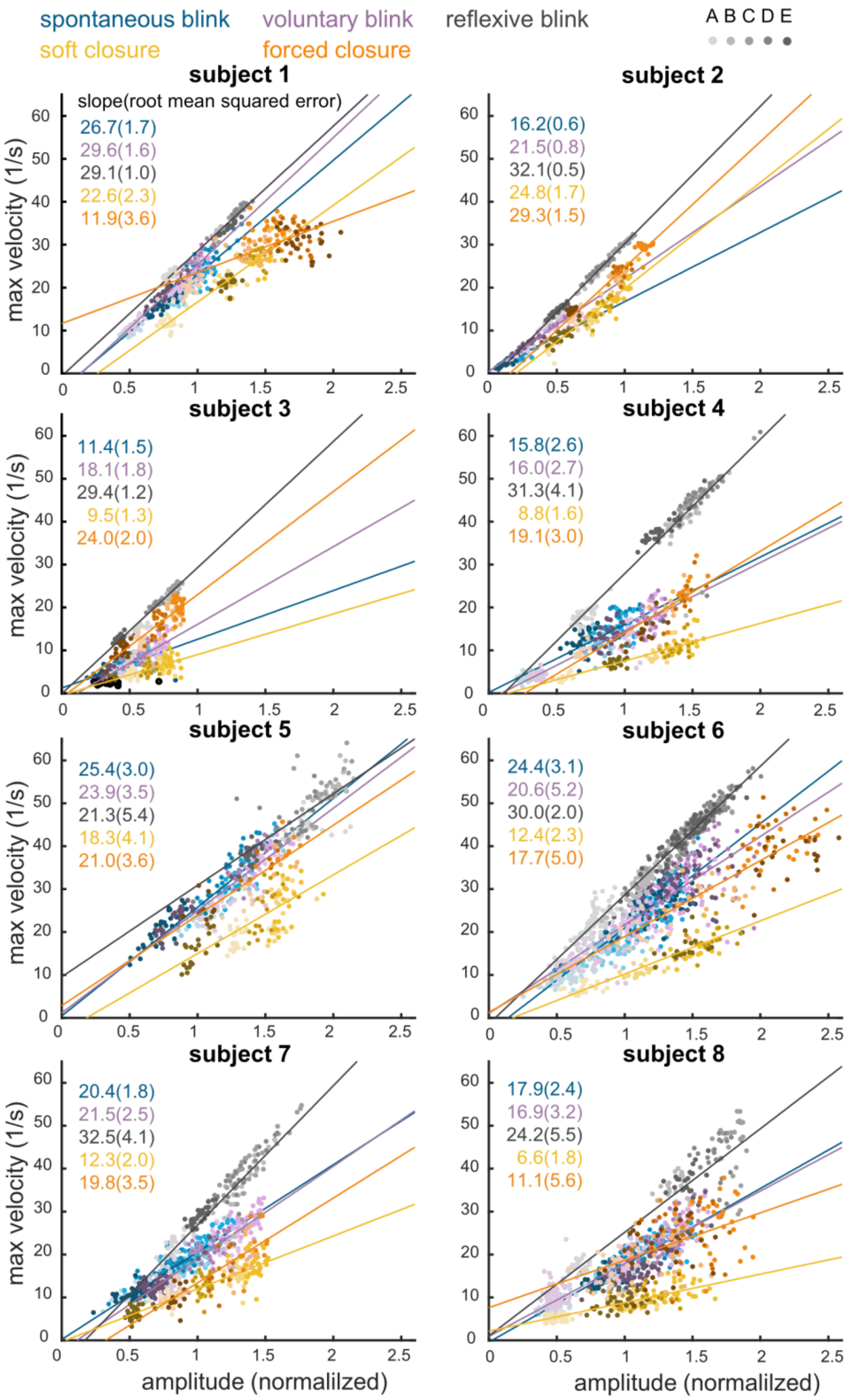

Figure S3. Main sequence analysis for each individual subject during the closing phase of different eyelid behaviors. Each point represents a single marker for a single trial. Linear regression lines are fit to all data from all upper markers (A-E) from all individual trials. Points are shaded according to marker position (A is lightest, E is darkest).

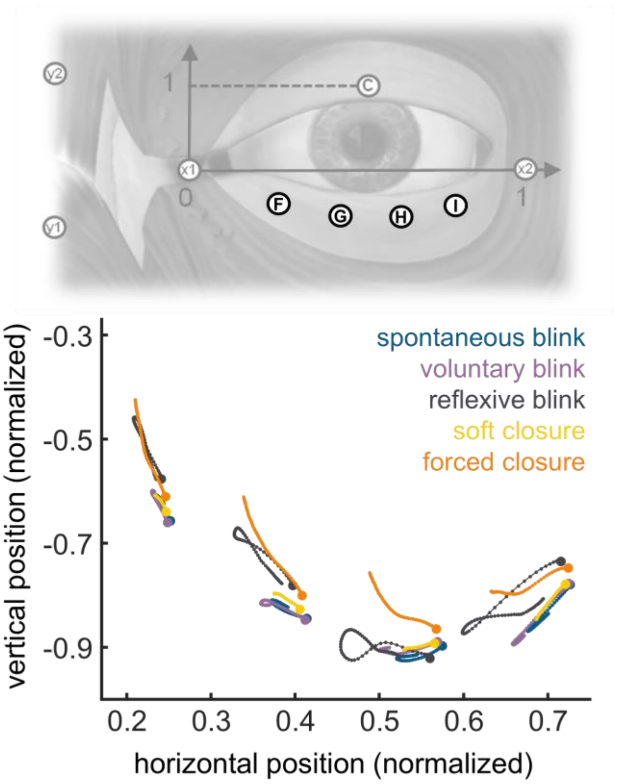

Figure S4. Intersubject average lower eyelid kinematics during different eyelid behaviors. Lines represent intersubject average kinematic trajectories. All motions start at the large colored dots. Each small colored dot represents a single motion capture frame; the time between dots is 2.5 ms.

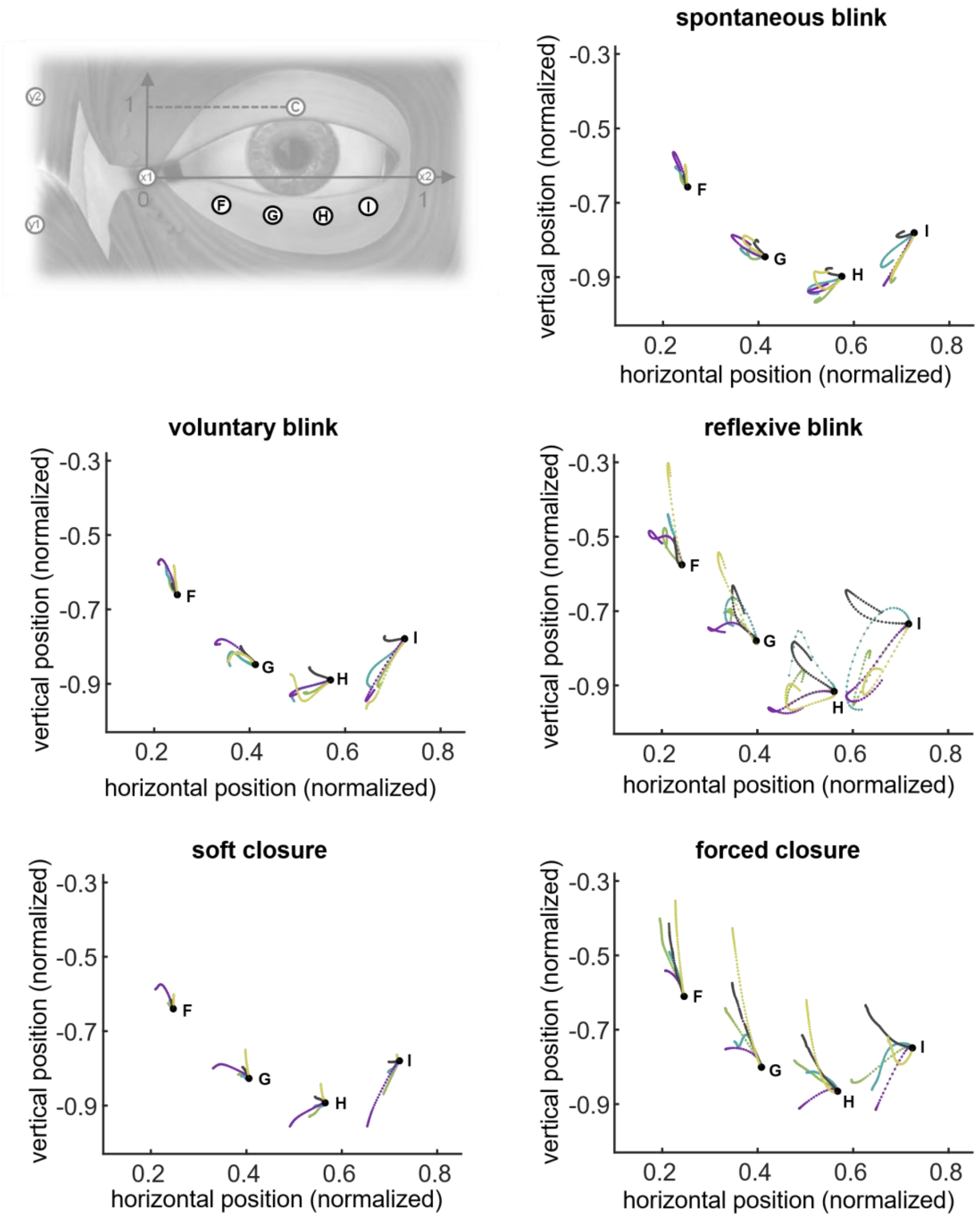

Figure S5. Individual subject lower eyelid kinematics during different eyelid behaviors. Lines represent intrasubject average kinematic trajectories. All motions start at the black dot; to facilitate comparison all trajectories are uniformly translated so that their starting point coincides with the inter-subject average starting point. Each colored dot represents a motion capture frame; the time between dots is 2.5 ms.

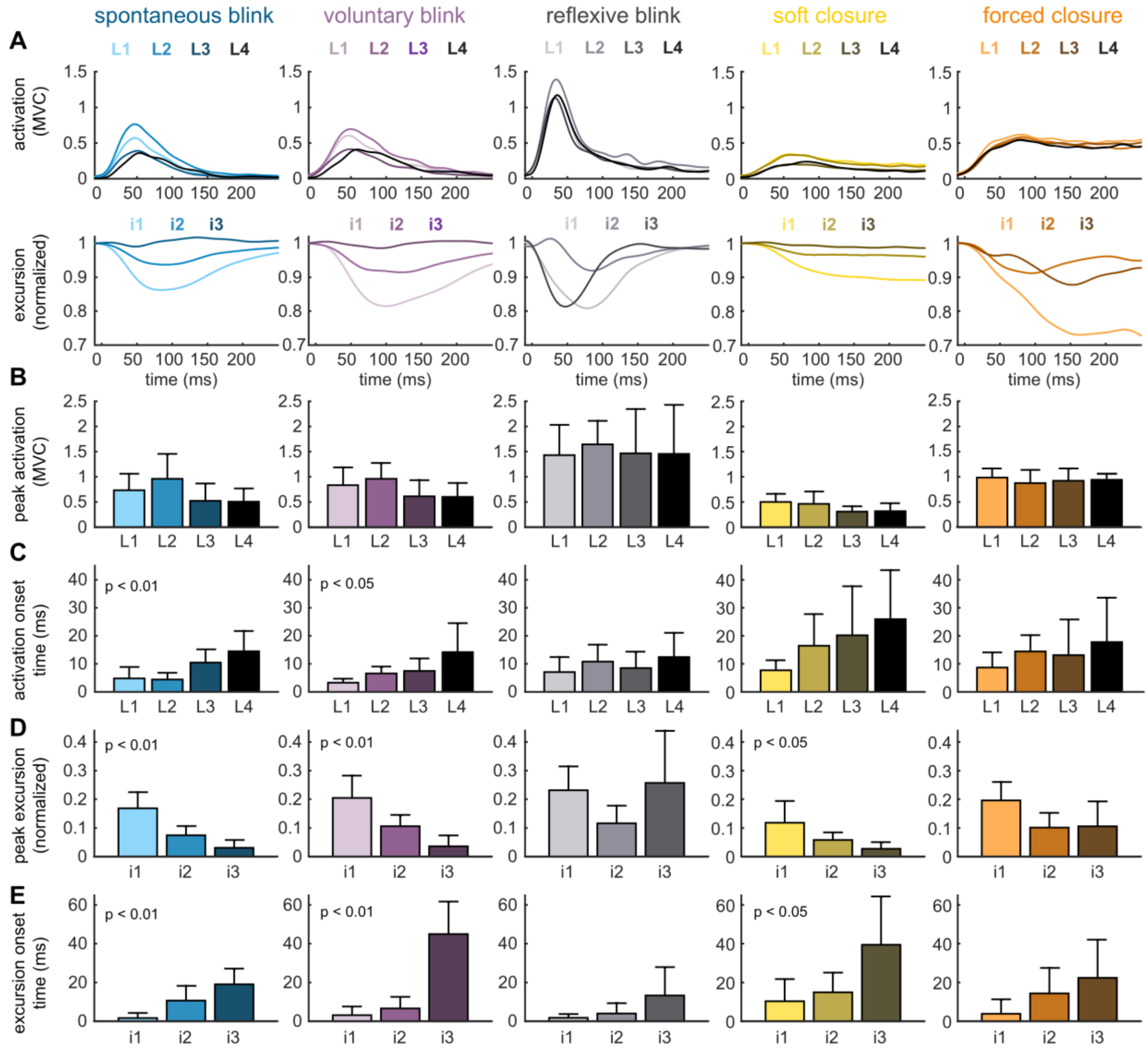

Figure S6. Segmental variation in activation and excursion across the lower pretarsal region of the OO muscle. Trajectories and bars show intersubject mean. Error bars show intersubject standard deviation. (**A**) Activation and excursion during each eyelid behavior. Activation values are normalized to the peak value of EMG from that electrode during forced closure. Excursion values are normalized to distance between markers with the eyelid open and at rest. (**B**) Peak activation in each electrode, normalized to the peak value of EMG from that electrode during forced closure. (**C**) Activation onset time, calculated relative to the first activation onset across the four electrodes in the lower pretarsal OO, for each trial. (**D**) Peak negative excursion, calculated based on inter-marker distances. (**E**) Excursion onset time, calculated relative to the first negative excursion onset across the three inter-marker distances on the lower eyelid margin, for each trial.

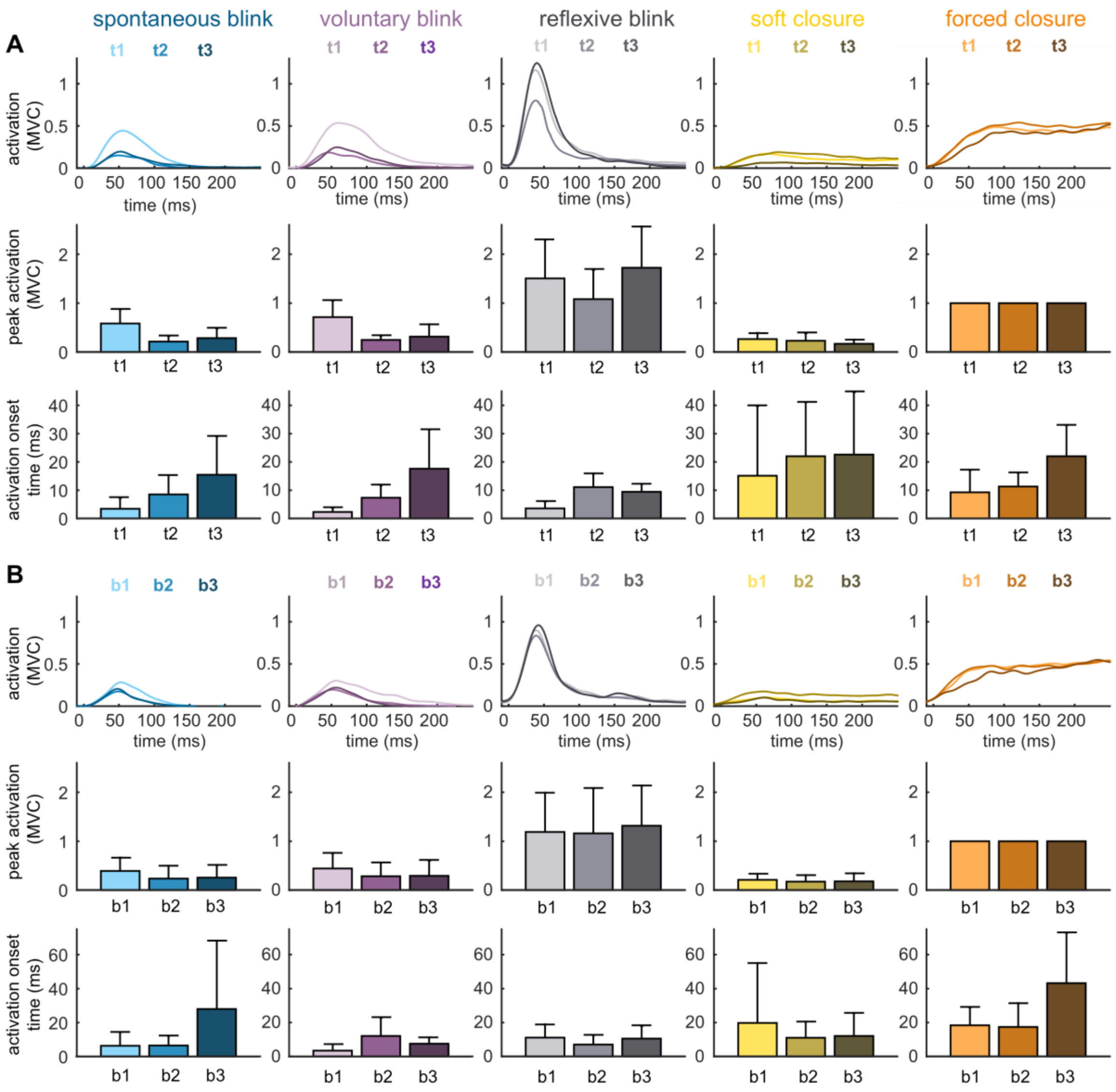

Figure S7. Segmental variation in activation across the (**A**) upper and (**B**) lower preseptal region of the OO muscle. Trajectories and bars show intersubject mean. Error bars show intersubject standard deviation. Activation and peak activation values are normalized to the peak value of EMG from that electrode during forced closure. Activation onset times are calculated relative to the first activation onset across the three electrodes in the region, for each trial.

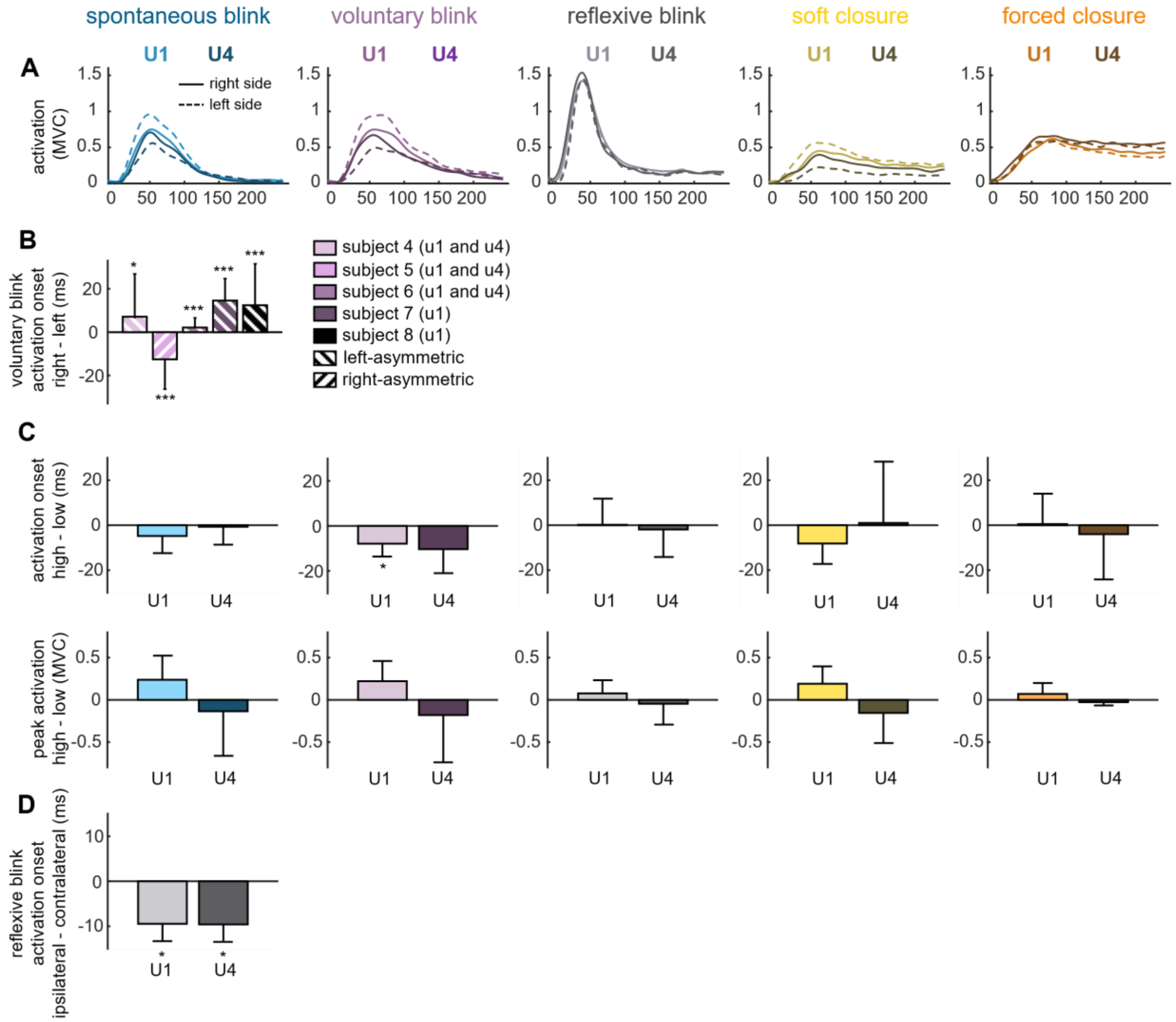

Figure S8. Differences in activation timing and intensity between sides of the face. Trajectories and bars show intersubject mean. Error bars show intersubject standard deviation. Statistical significance is indicated by asterisks (* $p < 0.05$, ** $p < 0.01$, *** $p < 0.001$.) (**A**) Activation at two different electrode locations on each side of the face. Activation values are normalized to the peak value of EMG from that electrode during forced closure. (**B**) Difference (right minus left) in activation onset during voluntary blinking. A positive value means that left side onset occurs earlier than right side onset; this is interpreted to mean that left side has higher motor neuron excitability (also described as left-asymmetric). Subjects 4, 5, and 6 had electrodes U1 and U4 on both sides of the face, and therefore both U1 and U4 were used for analysis. Subjects 7 and 8 had only U1 on both sides, and therefore only U1 was used for analysis. (**C**) Difference (higher excitability side minus lower excitability neuron side) in peak activation and activation onset time at each electrode location. (**D**) Difference (ipsilateral minus contralateral) in activation onset time during reflexive blink. Air puff stimulus was applied to the ipsilateral side to trigger the reflex. A negative value means that the ipsilateral side onset occurs earlier than the contralateral side.
